# Antimicrobial Coatings Based on a Photoreactive (Meth)acrylate Syrup and Ferulic Acid—The Effectiveness against *Staphylococcus epidermidis*

**DOI:** 10.3390/polym16172452

**Published:** 2024-08-29

**Authors:** Agnieszka Kowalczyk, Agata Kraśkiewicz, Agata Markowska-Szczupak, Krzysztof Kowalczyk

**Affiliations:** 1 Department of Chemical Organic Technology and Polymeric Materials, Faculty of Chemical Technology and Engineering, West Pomeranian University of Technology in Szczecin, 70-322 Szczecin, Poland; kkowalczyk@zut.edu.pl; 2 Department of Chemical and Process Engineering, Faculty of Chemical Technology and Engineering, West Pomeranian University of Technology in Szczecin, 71-065 Szczecin, Poland; agata.markowska@zut.edu.pl

**Keywords:** ferulic acid, (meth)acrylate, varnish, photopolymerization, antimicrobial coating

## Abstract

A novel photopolymerizable (meth)acrylate oligomer syrup modified with ferulic acid (FA) was verified as an antimicrobial coating binder against a biofilm of *Staphylococcus epidermidis*. A solution-free UV-LED-initiated photopolymerization process of aliphatic (meth)acrylates and styrene was performed to prepare the oligomer syrup. The influence of ferulic acid on the UV crosslinking process of the varnish coatings (kinetic studies using photo-DSC) as well as their chemical structure (FTIR) and mechanical (adhesion, hardness), optical (gloss, DOI parameter), and antibacterial properties against *S. epidermidis* were investigated. The photo-DSC results revealed that FA has a positive effect on reducing the early occurrence of slowing processes and has a favorable effect on the monomer conversion increment. It turned out, unexpectedly, that the more FA in the coating, the greater its adhesion to a glass substrate and hardness. The coating containing 0.9 wt. part of FA exhibited excellent antimicrobial properties against *S. epidermidis*, i.e., the bacterial number after 24 h was only 1.98 log CFU/mL. All the coatings showed relatively high hardness, gloss (>80 G.U.), and DOI parameter values (30–50 a.u.).

## 1. Introduction

Healthcare-associated infections (HAIs) are an important cause of morbidity and mortality in hospitalized patients. Many recent studies have suggested that environmental contamination plays an important role in the transmission of multidrug-resistant microorganisms, viruses, mycobacteria, and fungi [1]. One of the pathogens is *Staphylococcus epidermidis*. This is a well-known Gram-positive and aerobic bacterium, which is commonly found on the mucous membranes of the mouth and nose, and on the skin of humans and other mammals. Although it is classified as commensal organism, it is considered as an opportunistic pathogen. Currently, this species ranks third on the list of pathogens that cause nosocomial infections and are responsible for HAIs. Importantly, *S. epidermidis* is extremely difficult to treat due to biofilm formation and having genes for resistance to several antibiotics (e.g., rifampicin, gentamicin, and fluoroquinolones) [2]. *S. epidermidis* has been reported as being predominant among theatrical species, colonizing implanted devices with its major virulence factor of framing biofilms on various polymeric surfaces [3]. Generally, patients are a major source of microorganisms in the hospital environment. These microorganisms can survive in the hospital environment for long periods of time and can be detected in the air, water, and on surfaces. It is now accepted that targeted and frequent cleaning and the selection of appropriate equipment (including medical equipment such as hospital furniture) can reduce the bioburden in the healthcare environment and the associated risk of infections [4]. Studies over the past decade have indicated that bed frames and patient tables are microflora reservoirs and promote microorganisms’ survival [5]. The solution to the problem may be to coat solid surfaces (e.g., bed rails, hospital furniture) with biocidal paints or varnishes. Some reports have shown the antibiofilm activity against *S. epidermidis* of certain compounds such as mefenamic acid, acetaminophen, acetylsalicylic acid, carvacrol, 2-aminobenzemidazole or 3-indole acetonitrile [6]. Pilz at al. demonstrate that a multilayer ceramic-covered surface with a zirconium nitride topcoat showed less *S. epidermidis* biofilm formation than other surface materials used for orthopedic implants [7]. Ma et al. have developed a novel coating based on trimethylsilane as a monomer to coat the surfaces of stainless steel and a titanium alloy, which are widely used in implantable medical devices [8]. Skovdal et al. improved an ultra-dense poly(ethylene glycol)-based coating, significantly reducing the number of *S. epidermidis* bacteria adhering to titanium surfaces [9]. Antimicrobial coatings have a big role in controlling disease spread and preventing microbial colonization on surfaces [10,11]. Owing to the fact that polymeric materials are characterized by good mechanical properties, chemical stability, flexibility, and low costs, they have been widely used as antimicrobial coatings [12]. Additionally, the wide possibilities of polymer modification contribute to the design of thin films resistant to various environmental conditions (including a corrosive atmosphere) [13,14] or UV radiation resistance [15]. Among the different methods of production of polymer coatings, the UV-initiated radiation curing has lately become extremely important. The UV technology provides numerous advantages, i.e., high curing rates at room temperature, solvent-free formulations, and low emissions of volatile organic compounds (VOCs). The mentioned benefits make the UV technology economical and environmentally friendly [16,17,18,19].

It is worth noting that UV-curable formulations usually contain functionalized acrylate oligomers/prepolymers, a photoinitiator(s), and a reactive diluent(s) [20]. Various types of acrylate-based resins (e.g., polyurethane, epoxy, polyether, or polyester acrylates) allow the preparation of materials with specific properties [21]. Bio-polymers (such as chitosan [22] and peptides [10]) as well as graphene [23] and compounds containing metals (Ag [24], Au [25], Zn [26], and Cu [27]) or TiO_2_ particles [28] are frequently used for improving the antimicrobial properties of conventional polymeric coatings. Additionally, the interest in the properties of natural compounds with health-promoting properties is constantly increasing. Examples of such compounds are ferulic acid (FA) or (E)-3-(4-hydroxy-3-meth oxyphenyl)prop-2-enoic acid (C_10_H_10_O_4_), belonging to the phenolic acids. FA was isolated in 1866 from the *Ferula foetida* plant, which gave it its name. Nevertheless, it can be extracted from cereals [29], sugar beet [30], or residues and by-products from the pine species [31]. In natural products, FA occurs in the form of a monomer, dimer, free oligomer, or polymers [32]. Nowadays, FA is obtained through chemical or biotechnology methods [33]. It was proved that it has antioxidant, antidiabetic, hepatoprotective, anti-atherosclerotic, neuroprotective, anticarcinogenic, and antibacterial properties [34]. This is due to the chemical reactivity of FA, which can involve donating an electron or hydrogen atom, removing free radicals, and interrupting oxidative reactions [35]. It is known that FA has strong antibacterial properties against Gram-negative bacteria (e.g., *Escherichia coli, Klebsiella pneumoniae Citrobacter koseri, Pseudomonas aeruginosa, Helicobacter pylori*, and *Shigella sonnei*) and selected Gram-positive bacteria [36]. The effectiveness of FA and its derivatives against Gram-positive *S. aureus* has been recently proven [37]. Moreover, its resistance to bacterial colonization and the additional unique properties of ferulic acid make it commonly used in the pharmaceutical and cosmetic industries [38]. Additionally, the chemical structure of FA provides various application prospects. The presence of phenol and carboxylic groups allows a wide range of modifications [39], whereas the content of the double bonds enables its potential for use as a replacement in poly(ethylene terephthalate) [40], the starting building block for polyurethanes, epoxides, as well as phenolic resins [41]. Nevertheless, applications of FA in radical photopolymerization are little described. Pezzana et al. reported the allylation process of FA to achieve mono- and bifunctional allyl monomers to a UV-initiated thiol-ene polymerization. Notwithstanding this, the authors do not present the antimicrobial properties of the obtained protective coatings [42]. To the best of the authors’ knowledge, there is only one article describing the direct copolymerization of FA with methacrylic acid to obtain a useful material with antioxidant and antifungal properties. For this purpose, a one-step radical polymerization using water-soluble redox initiators was performed [43].

In this paper, we describe the use of low concentrations of ferulic acid in varnish coatings, enabling their radical UV crosslinking process and providing antimicrobial properties. The varnish coatings are based on a (meth)acrylate syrup prepared via a solvent-free UV-LED-initiated photopolymerization of a few aliphatic (meth)acrylates and styrene. In addition, the coating compositions contained a multifunctional crosslinking monomer and hydroxyl alkyl phenyl acetophenone (HAP) as a photoinitiator. The influence of ferulic acid on the UV crosslinking process of the varnish coatings and their adhesion, hardness, gloss, and antimicrobial properties were investigated.

## 2. Materials and Methods

### 2.1. Materials

**Figure 1:**
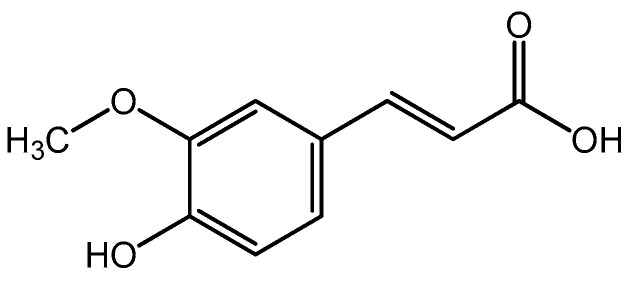
Chemical structure of ferulic acid.

The following materials were used: n-butyl acrylate (BA; BASF, Ludwigshafen, Germany); methyl methacrylate (MMA; Sigma Aldrich, St. Louis, MI, USA); 2-hydroxyethyl acrylate (HEA; Across Organics, Geel, Belgium); styrene (STY; Sigma Aldrich, St. Louis, MI, USA); bis(2,4,6-trimethylbenzoyl)-phenylphosphine oxide (a radical photoinitiator, APO; Omnirad 819, IGM Resins, Waalkwijk, The Netherlands); pentaerythritol triacrylate (a multifunctional crosslinking monomer, PETIA; Allnex, Drogenbos, Belgium), 1-hydroxycyclohexylphenyl ketone (a radical photoinitiator, HAP; Omnirad 184, IGM Resins, Waalkwijk, The Netherlands), and ferulic acid (FA; Sigma-Aldrich, St. Louis, MI, USA) (Figure 1).

### 2.2. Preparation of the (Meth)acrylate Syrup

**Figure 2:**
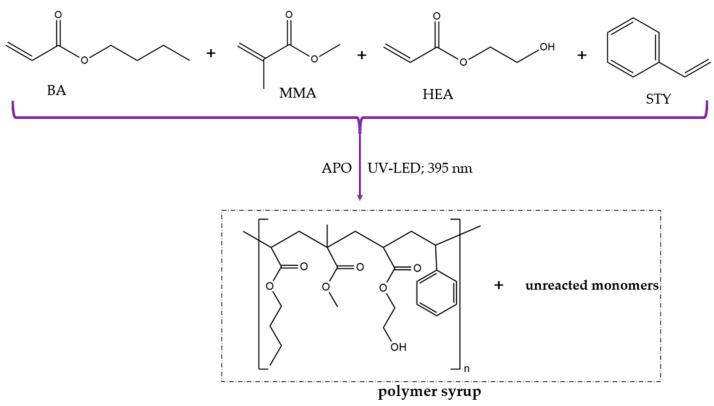
The preparation scheme of the (meth)acrylate syrup by free radical bulk photopolymerization.

The (meth)acrylate syrup (MAS) was synthesized via the solvent-free UV-induced polymerization process of a monomer mixture (BA, MMA, HEA, and STY) in the presence of a radical UV photoinitiator (APO) (Figure 2). The photopolymerization was conducted at 50 °C in a glass reactor (250 mL) equipped with a thermocouple, a mechanical stirrer (300 rpm), a water cooler, and a capillary dosing an inert gas (Ar). The reaction mixture was irradiated by UV-LED stripes (λ = 390 ± 5 nm; MEiSSA, Warsaw, Poland) for 40 min. The composition of the UV-polymerized mixture is presented in Table 1.

### 2.3. Preparation of the Varnish Compositions and Coatings

**Figure 3:**
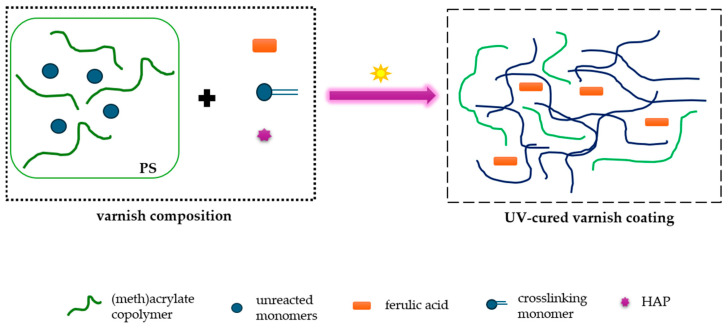
The preparation scheme of the UV-cured varnish coatings containing ferulic acid.

The UV-photocurable varnish compositions were prepared by mechanical mixing of the MAS, the PETIA monomer, the HAP UV photoinitiator, and ferulic acid, as indicated in Figure 3 and Table 2. The systems were applied onto glass substrates (24 h after mixing the ingredients) using a gap applicator (60 µm). The prepared varnish coatings were UV-irradiated with the medium-pressure mercury lamp (UV-ABC, Hönle UV-Technology, Gräfelfing, Germany). The total UV radiation dose was 6 J/cm^2^.

### 2.4. Characterization of the (Meth)acrylate Syrup

The dynamic viscosity (η) of MAS was measured at 25 °C using the DV-II Pro Extra viscometer (spindle #7; 50 rpm; Brookfield, New York, NY, USA). The solids content (SC) was checked utilizing a thermobalance (Radwag, Warsaw, Poland): samples (ca. 2 g) were heated in aluminum pans at 105 °C for 4 h. The molecular masses (M_w_, M_n_) and polydispersity (PDI) of copolymers (the solids of MAS) were characterized by gel permeation chromatography (GPC). The GPC apparatus contained the refractive index detector (Merck Lachrom RI L-7490), pump (Merck Hitachi Liquid Chromatography L-7100), interface (Merck Hitachi Liquid Chromatography D-7000), and the Shodex OHpak SB-806M MQ column with the Shodex OHpak SB-G precolumn. The GPC tests were performed using the polystyrene standards (Fluka and Polymer Standards Service GmbH, Mainz, Germany) and tetrahydrofuran.

### 2.5. Kinetic Studies of the UV Curing Process of the Varnish Compositions 

The kinetics of the photocuring process of the coating compositions were characterized using the differential scanning calorimeter with the UV equipment (Q100, TA Instruments, New Castle, DE, USA). The varnish compositions (ca. 5 mg, Table 2) were UV-irradiated (280–420 nm) at an intensity of 500 mW/cm^2^ in a nitrogen atmosphere (the same conditions were applied during the UV curing process with a UV-ABC-type medium-pressure mercury lamp). The photopolymerization rate (*R_p_*, *s*^−1^), conversion of double bonds (p,%), and the photoinitiation index (*I_p_*; *s*^−2^), were calculated according to Equations (1)–(3), respectively. Additionally, the evaluation of slowing processes as the ratio of double bond conversion (at the point corresponding to the maximum reaction rate) to the total conversion of the monomers (*p_Rp_^max^/p^f^*) was studied.

(1) $$R_{p}=\frac{\left(\frac{dH}{dt}\right)}{H_{0}}\left[s^{-1}\right]$$

(2) $$p=\frac{∆H_{t}}{∆H_{0}}*100\;\left[\%\right]$$

(3) $$I_{p}=\frac{R_{p}^{max}}{t_{max}}\;\left[s^{-2}\right]$$

where *dH*/*dt* is the recorded heat flow during UV irradiation, *H*_0_ is the theoretical heat value for the complete degree of conversion (∆*H* = 86.2 kJ/mol for acrylates, ∆*H* = 54.8 kJ/mol for methacrylates, and ∆*H* = 67.4 kJ/mol for styrene), ∆*H_t_* is the reaction heat released at time *t*, and *t_max_* is the time taken to reach the maximum polymerization rate [44].

### 2.6. Structural Characteristics of the UV-Cured Varnish Coatings

A functional group analysis of the UV-cured varnish coatings containing ferulic acid was carried out using the Fourier-transform infrared spectroscope with ATR accessories (Nexus Euro, Thermo Scientific, Waltham, MA, USA). A double bond peak at 1635 cm^−1^ (in acrylates) and a double bond at 1621 cm^−1^ (in ferulic acid) [45] were investigated.

### 2.7. Mechanical and Optical Characteristics of the UV-Cured Varnish Coatings

The pendulum hardness of the varnish coatings cured on a glass substrate was tested using the König pendulum (AWS-5, Dozafil, Warsaw, Poland) according to ISO 1522 [46] The pull-off adhesion of the coatings to this substrate was measured according to ISO 4624 [47] using the pull-off adhesion apparatus with Ø20 mm aluminum dollies (Elcometer 510; Elcometer, Manchester, UK) and a two-component epoxy adhesive (Loctite, Rocky Hill, CT, USA). The gloss (20°) and distinctness of the image parameters (DOI) of the varnish coatings cured on white paper panels (WDX Plain White Cards, Leneta Company, Mahwah, NJ, Leneta, USA) were determined by ISO 2813 [48] and ASTM D5767 [49], respectively. The measurements were realized using the IQ20/60/85 device (Rhopoint Instruments, St Leonards, UK). The values of the tested parameters were calculated by averaging five measurements of each sample.

### 2.8. Antibacterial Properties of the UV-Cured Varnish Coatings

The antibacterial properties of the UV-cured varnish coatings were determined according to ASTM E2149-20 [50]. *Staphylococcus epidermidis* (ATCC 49461) was used as a model strain which was grown in Brain Heart Infusion Broth (BHI) (Biocorp, Warsaw, Poland). The overnight cultures of the bacteria were transferred to a TBS buffer (50 mM Tris-Cl; 150 mM NaCl pH 7.5; Chemland; Stargard, Poland). The cultures were diluted with buffer until the final concentration of the bacteria was approx. 1.0 × 10^6^. The varnish coatings were tested according to a modified standard test method for determining the antimicrobial activity of antimicrobial agents under dynamic contact conditions. Control glass plates and glass plates (1 × 1 cm) covered by the coatings were sterilized under a UV-C lamp for 15 min and placed into a sterile buffer in 250 mL screw-cap Erlenmeyer flasks. Then, a 50 ± 0.5 mL dose of the working dilution of the prepared bacterial inoculum was added to the flask. The series of flasks was shaken using a wrist-action shaker (at maximum stroke) at 37 °C for 1 h, and samples (0.5 mL) were collected after 0.5, 1, and 24 h. Bacterial concentration was measured at the “0” time and during the experiments by serial dilutions and standard plate-count techniques in triplicate. The Brain Heart Infusion Agar (Biocorp, Warsaw, Poland) was used. All the Petri dishes were incubated at 37 ± 2 °C for 24 h. The visible bacteria colonies were counted and reported as colony-forming units per milliliter (CFU/mL). The survivability of the bacteria (%) was calculated.

## 3. Results and Discussion

### 3.1. The Physicochemical Properties of the (Meth)acrylate Syrup and Copolymer

The product of the solvent-free UV-LED-initiated photopolymerization was a solution of a linear copolymer in unreacted monomers (MAS). The selected properties of the prepared syrup, i.e., the dynamic viscosity (ƞ), the solids content (SC) as well as the molecular masses (Mn; Mw) and the polydispersity index (PDI) of the (meth)acrylate copolymer are presented in Table 3.

In our previous publications, we have reported that free radical bulk photopolymerization (FRBP) is a particularly convenient method for obtaining adhesive binders [51,52] in a short time (tens of minutes) and in an energy-efficient way (using UV-LEDs). In this article, we tested the method during the preparation of varnish binders. The choice of monomers is justified. Namely, the main monomers are n-butyl acrylate and methyl methacrylate. The former imparts flexibility as well as atmospheric and water resistance to the coatings, while the latter increases their hardness. The HEA monomer was used as a functional monomer with a hydroxyl group that provides good adhesion to various substrates. Styrene improves gloss and hardness as well. As can be seen, the prepared syrup contains ca. 50% of (meth)acrylate oligomers (due to small molecular weight values) with relatively low polydispersity (3.6). The syrup’s viscosity was relatively low and suitable (4.6 Pa∙s) for further modification using the crosslinking monomer, photoinitiator, and ferulic acid. As presented in Figure 3, during the UV curing process of the varnish coatings, the unreacted monomers in the syrup (ca. 50%) and the crosslinking monomer (PETIA) may create a polymer network. The result is a semi-IPN polymer blend containing a linear methacrylate copolymer and a crosslinked polyacrylate structure. We postulate that the semi-IPN polymer matrix includes ferulic acid which is not covalently bound to the polymeric system.

### 3.2. Kinetic Studies of UV Curing of the Varnish Composition

**Figure 4:**
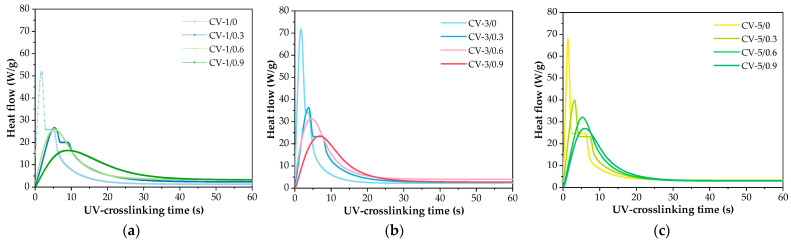
The kinetics curves of UV crosslinking processes of the varnish binders with various contents of ferulic acid and the HAP photoinitiator (1 wt. part (**a**), 3 wt. parts (**b**), and 5 wt. parts of HAP (**c**)).

**Figure 5:**
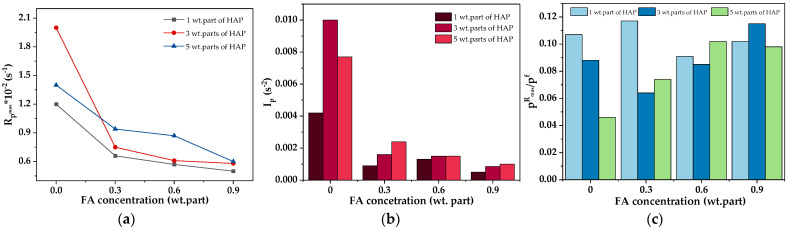
The influence of ferulic acid concentration on the maximum reaction rate (*R_p_^max^*) (**a**), the photoinitiation index (**b**), and the slowing process evaluation parameter (**c**) of the varnish coatings.

Considering the kinetics of the photocrosslinking process of the varnish compositions, a key aspect is the type of monomers, photoinitiator, and the presence of the phenolic compound (FA). The results of the NMR analysis of the syrup were presented in our previous publication [53] (MAS was tested as a reference sample (P-0) for systems modified with organophosphorus compounds). According to the NMR data, MAS contains unreacted BA (53 wt.%), MMA (22 wt.%), HEA (20 wt.%), and STY (5 wt.%). These outcomes (and the concentration of the added PETIA monomer) were taken into account in the calculations. The results of the kinetic studies of the varnish compositions with different contents of the HAP photoinitiator (1, 3 or 5 wt. parts) as well as ferulic acid (0.3, 0.6 or 0.9 wt. part per 100 wt. parts of MAS) are presented in Figure 4. Generally, the applied HAP photoinitiator generates two types of radicals, i.e., a benzoyl radical and a hydroxycyclohexyl radical, and they both react with the (meth)acrylate double bonds [54]. As mentioned earlier, ferulic acid belongs to the group of antioxidants. This is due to the possibility of forming a stable phenoxy radical. Generally, the rupture of the O-H bond in phenols with the formation of radicals may occur in the presence of ionizing radiation (including ultraviolet radiation) or by contact with free radicals. As a result, a phenoxy radical is formed which is stabilized by the influence of the aromatic ring and its substituents. In biotechnology, FA is an effective compound to combat scavenging and stop free radicals’ chain reactions. Our research shows that FA slows down the UV crosslinking process of the varnish coating compositions. The higher the FA concentration in the system, the significantly lower the *R_p_^max^* parameter values (Figure 5a). In addition, the slowing down of the process is evidenced by the values of *t_Rp_^max^* (Table 4). In the case of the FA-free systems, the *t_Rp_^max^* values vary from 1.8 s to 2.8 s (for 5 and 1 wt. parts of HAP, respectively). In contrast, the *t_Rp_^max^* values for the FA-based systems changed from 3.8 to even 9.4 s (the higher values for the higher HAP concentrations).

Moreover, it was shown that FA significantly reduces the initiation ability of the process because I_p_ values are lower for systems with FA than for the reference sample (I_p_ decreases with increasing FA content; Figure 5b). This is related to the reaction of benzoyl and hydroxycyclohexyl radicals (from HAP) with ferulic acid (i.e., abstraction of the hydrogen atom from the hydroxyl group of the acid and formation of a phenoxy radical). This phenomenon deactivates a certain number of radicals initiating the photopolymerization process (its rate is decreased). Nonetheless, it was found that FA has no negative effect on monomer conversion. These results are presented in Table 4. It should be noted that the monomer conversion was relatively large (67–81%); the lowest value was recorded for the CV-5/0 sample, while the highest was for CV-1/0.9. The result indicates that FA increases the conversion of the monomers (the increment of 3% was observed for samples containing 0.9 wt. part of the acid). Additionally, the photo-DSC results revealed that FA has a positive influence on the reduction of the early occurrence of slowing processes, such as chain transfer reactions (Figure 5c)—the low values of the p^R^_max_/p^f^ ratio reveal this phenomenon. It was proved that slowing down processes occur more intensively in the system containing 5 wt. parts of HAP (without FA), which is associated with a higher number of macroradicals. 

In contrast, the higher the concentration of the acid, the higher the value of this ratio (FA has a positive influence on the photocrosslinking process). It confirms the deactivation of a certain number of primary radicals by acid molecules, which varies the amount of macroradicals and results in increased monomer conversion.

### 3.3. Structural Characterization of UV-Cured Varnish Coatings

**Figure 6:**
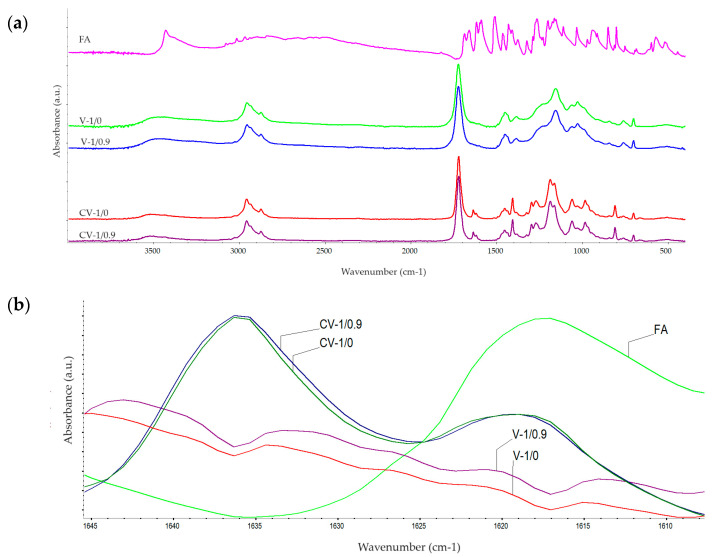
The FTIR spectra for ferulic acid and the selected uncured and UV-cured varnish compositions (full wavenumber range (**a**) and a narrow range (**b**)).

FTIR spectroscopy was used to study the curing reaction of the varnish compositions via detection of the presence/intensity of the monomer double bonds; the FTIR spectra for FA and selected uncured and cured varnish compositions are shown in Figure 6. The observed trend was similar for all the tested systems. The intensities of the band at 1635 cm^−1^ (corresponding to the C=C bonds of the (meth)acrylate monomers and styrene) were the same for the uncured FA-free and FA-based compositions and they were reduced to similar values after the curing process. On the other hand, the values of the peak at 1621 cm^−1^ (the C=C bonds in FA and the PETIA monomer) were different for the cured reference and modified varnishes. Probably, it means that FA does not polymerize with other unsaturated components of the varnish systems. 

### 3.4. Mechanical and Optical Properties of the UV-Cured Varnish Coatings

**Figure 7:**
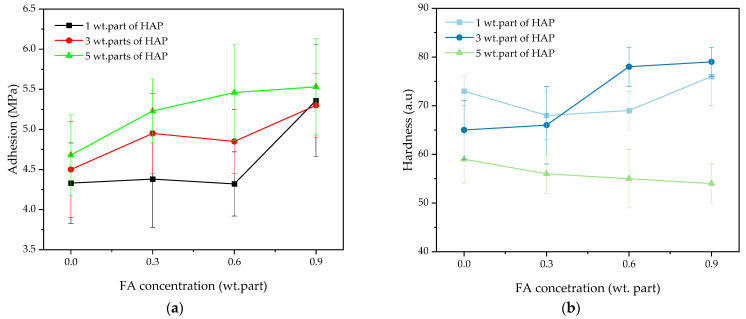
The adhesion to a glass substrate (**a**) and hardness (**b**) of the UV-cured varnish coatings.

The aim of the studies was to determine the influence of FA on selected features of the UV-cured varnish compositions. The values of adhesion to the glass substrate and hardness of the coatings are presented in Figure 7.

Generally, the coatings were characterized by high adhesion (4.2–5.5 MPa); its values increased with increasing concentrations of the HAP photoinitiator and ferulic acid. As shown by the photo-DSC and FTIR results, the monomer conversion was reduced by a higher HAP dose, and it slightly increased with increasing acid content. It means that the crosslinking density is lower in the samples with the higher HAP concentration. For this reason, the adhesion was higher due to the easier access of the polar groups from the varnish matrix (-OH introduced by HEA, PETIA, and FA) to the surface layer, i.e., the easier formation of hydrogen bonds with HO-Si- groups from the glass substrate. Nevertheless, the best results were obtained for the systems containing 0.9 wt. part of FA (regardless of the HAP content). In contrast, the opposite tendency was observed for the pendulum hardness parameter. The lowest values (53–58 a.u.) were noted for the coatings with 5 wt. parts of HAP and they were also associated with low crosslinking densities of the systems. The best hardness results were registered for the V-3/0.6 and V-1/0.9 coatings (78 and 79 a.u., respectively). 

**Figure 8:**
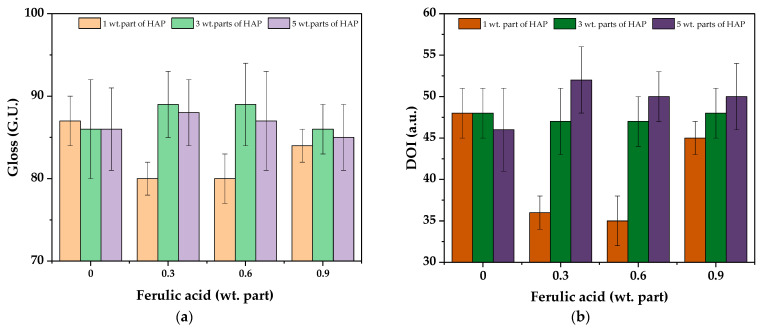
The gloss (**a**) and DOI values (**b**) for the UV-cured varnish coatings.

The gloss and the DOI values for the cured varnish coatings are presented in Figure 8. The samples were characterized by relatively high gloss (ca. 85–89 G.U.), and this parameter was not markedly affected by the HAP and ferulic acid concentration; slightly lower results were recorded only for the V-1/0.3 and V-1/0.6 samples (probably due to the higher crosslinking density of these systems). Similar trends were observed for the DOI results. For these samples, DOI was slightly lower (ca. 36 a.u.) than for the others. On the other hand, the parameter values were generally higher for the samples containing FA and 5 wt. parts of the HAP photoinitiator. This feature (DOI) characterizes the sharpness/clarity of an image created by the reflection of an object surface. The observed DOI increment (for V-5-type systems) indicates that their surfaces have become more smooth. It correlates with the monomer conversion results (based on the photo-DSC test), which indicate the lower crosslinking density of these samples.

### 3.5. Antimicrobial Properties of the UV-Cured Varnish Coatings

**Figure 9:**
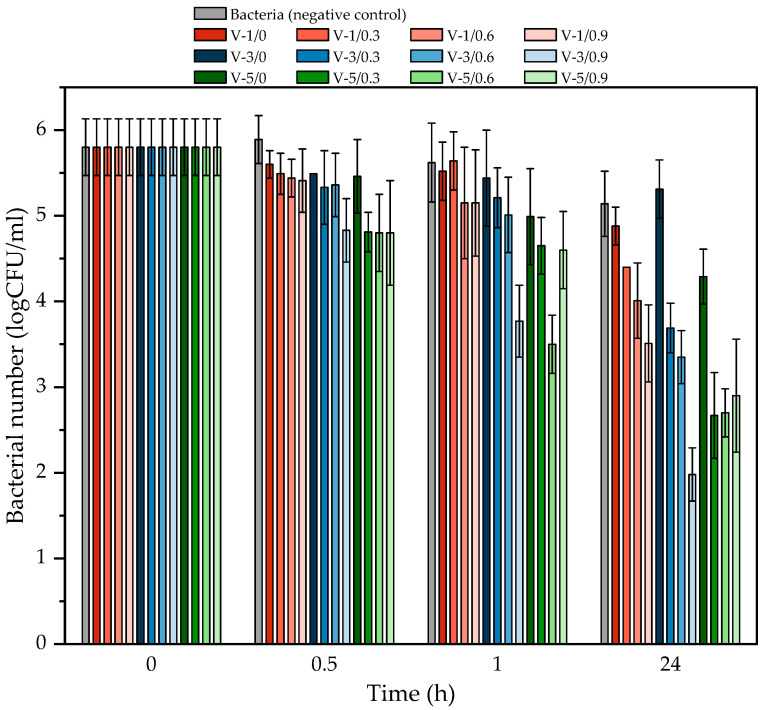
The reduction of *Staphylococcus epidermidis* after contact with the UV-cured varnish coatings.

The antimicrobial test results are presented in Figure 9. According to the existing EPA standards, a reduction greater than or equal to 6-fold logarithmic (≥6 log) in <10 min is needed to claim that a coating has excellent antimicrobial properties [55]. In the present study, we obtained twelve coatings with different contents of the photoinitiator and ferulic acid. In relation to the EPA standards, the obtained coatings exhibited a moderate antibacterial activity against *Staphylococcus epidermidis* (<1.5 log in 30 min); however, this feature (after 24 h) was already satisfactory. Generally, the best results were achieved for V-3/0.9 (3.8 log in 24 h) and for the V-5/0.3, V-5/0.6, and V-5/0.9 samples (ca. 3 log in 24 h). A reduction in the number of bacteria >3 log indicates the removal of 99% of the bacteria. As can be seen, this excellent reduction in the number of bacteria was achieved for samples with the lower monomer conversion values (and therefore crosslinking density) because the FA molecules—trapped in the semi-IPN polymer network—can more easily penetrate the top coating and interact with the *S. epidermidis* anchored on its surface. Additionally, the surfaces of the V-5-type varnish coatings modified with FA are smoother (as indicated by the DOI parameter), thus there are fewer surface defects which reduces the adhesion of *S. epidermidis* to these surfaces. It was demonstrated by Pinheiro et al. [56] that lipidsoluble substances (including FA) may increase permeability of the bacterial outer membrane, tipping the balance of the in and the out and facilitating the transport of molecules in cells. It can be essential for the penetration and distribution of an antibacterial agent, e.g., detergents and antibiotics. An accelerated lipid peroxidation process in cytoplasmic membranes is also possible due to the affinity of FA for the bacterial membranes. These changes in membrane integrity may be a significant cause of cell death.

We postulate that the good antibacterial activity of the varnish coatings was observed only after 24 h because during this period, more FA molecules migrated from the polymer network to the surface. Nevertheless, in each case, the better results were noted for the FA-based samples than for the reference samples (i.e., V-1/0, V-3/0, and V-5/0). 

## 4. Conclusions

New antimicrobial polymeric varnish coatings were prepared via the UV crosslinking process of a (meth)acrylic/styrene copolymer syrup modified with a crosslinking monomer (PETIA), a radical photoinitiator (HAP), and ferulic acid (FA). Mechanical and optical properties as well as antibacterial activity (against *Staphylococcus epidermidis*) of the coatings were tested. Moreover, the influence of the acid on the kinetics of the photocrosslinking process was investigated. The highest antimicrobial activity was demonstrated by the coating after 24 h and depended on FA and HAP concentrations; the best results were registered for the systems containing 0.9 wt. part and 3 wt. parts of these components, respectively (99% reduction in bacteria). Arguably, it resulted from the limited crosslinking density of the polymer network and the ability of the acid to migrate to the surface layer of the coatings. Although FA is known as an antioxidant, photo-DSC studies revealed its beneficial effect on the photocrosslinking process (FA increases monomer conversion and inhibits retarding phenomena). This acid addition positively influences the adhesion of the varnish coatings to a glass substrate and does not deteriorate their gloss or DOI parameter values.

## Figures and Tables

**Table 1:** The components of the photoreactive (meth)acrylate syrup.

(Meth)acrylate Syrup	Monomers [wt. parts]				APO [wt. part]
	BA	MMA	HEA	STY	
MAS	40	35	15	10	0.75

**Table 2:** The composition of the UV-photocurable varnishes.

Varnish Symbol	Components [wt. parts]			
	MAS	PETIA	HAP	FA
CV-1/0	100	15	1	0
CV-1/0.3				0.3
CV-1/0.6				0.6
CV-1/0.9				0.9
CV-3/0			3	0
CV-3/0.3				0.3
CV-3/0.6				0.6
CV-3/0.9				0.9
CV-5/0			5	0
CV-5/0.3				0.3
CV-5/0.6				0.6
CV-5/0.9				0.9

**Table 3:** The properties of the (meth)acrylate syrup and (meth)acrylate copolymer.

Syrup		Copolymer		
SC (%)	ƞ [Pa·s]	M_n_ [g/mol]	M_w_ [g/mol]	PDI [a.u.]
51	4.6	15,000	56,000	3.7

**Table 4:** The characteristic parameters of the UV crosslinking process of the varnish compositions.

HAP (wt. part)	1				3				5			
FA (wt. part)	0	0.3	0.6	0.9	0	0.3	0.6	0.9	0	0.3	0.6	0.9
Conversion (%)	78	74	77	81	72	71	72	75	67	68	70	70
*t_Rp_^max^* (s)	2.8	6.1	5.7	9.4	2.1	4.0	5.5	7.0	1.8	3.8	5.6	6.0

## Data Availability

The original contributions presented in the study are included in the article, further inquiries can be directed to the corresponding authors.
